# Causes and Predictors of In-Hospital Mortality in Patients Admitted with or for Heart Failure at a Tertiary Hospital in Brazil

**DOI:** 10.5935/abc.20170136

**Published:** 2017-10

**Authors:** André Wajner, Priccila Zuchinali, Vírgilio Olsen, Carisi A. Polanczyk, Luis Eduardo Rohde

**Affiliations:** 1 Hospital Nossa Senhora da Conceição, Porto Alegre, RS – Brazil; 2 Hospital de Clínicas de Porto Alegre, Porto Alegre, RS – Brazil; 3 Programa de Pós Graduação em Cardiologia e Ciências Cardiovasculares da FAMED/UFRGS, RS – Brazil

**Keywords:** Cardiovascular Diseases, Heart Failure, Hospital Mortality, Demographic Aging, Hospitals, Public

## Abstract

**Background:**

Although heart failure (HF) has high morbidity and mortality, studies in Latin America on causes and predictors of in-hospital mortality are scarce. We also do not know the evolution of patients with compensated HF hospitalized for other reasons.

**Objective:**

To identify causes and predictors of in-hospital mortality in patients hospitalized for acute decompensated HF (ADHF), compared to those with HF and admitted to the hospital for non-HF related causes (NDHF).

**Methods:**

Historical cohort of patients hospitalized in a public tertiary hospital in Brazil with a diagnosis of HF identified by the Charlson Comorbidity Index (CCI).

**Results:**

A total of 2056 patients hospitalized between January 2009 and December 2010 (51% men, median age of 71 years, length of stay of 15 days) were evaluated. There were 17.6% of deaths during hospitalization, of which 58.4% were non-cardiovascular (63.6% NDHF vs 47.4% ADHF, p = 0.004). Infectious causes were responsible for most of the deaths and only 21.6% of the deaths were attributed to HF. The independent predictors of in-hospital mortality were similar between the groups and included: age, length of stay, elevated potassium, clinical comorbidities, and CCI. Renal insufficiency was the most relevant predictor in both groups.

**Conclusion:**

Patients hospitalized with HF have high in-hospital mortality, regardless of the primary reason for hospitalization. Few deaths are directly attributed to HF; Age, renal function and levels of serum potassium, length of stay, comorbid burden and CCI were independent predictors of in-hospital death in a Brazilian tertiary hospital.

## Introduction

Despite the decline in many cardiovascular diseases, there is a stable or increased prevalence of heart failure (HF) in the world and in Brazil, which is probably due to population aging associated with an increase in survival in patients with cardiovascular diseases.^1^ Despite the great progress in its treatment, HF remains one of the main causes of hospitalization in several countries and is associated with high rates of morbidity and mortality.^2^ Even with optimized therapy, estimates account for a 4-year mortality rate of 40%,^3^ with a reduction in quality of life and prognosis when compared, for example, with various neoplasias.^2^

Observational studies in several countries have shown that after a hospitalization due to decompensated HF, significant changes occur in the natural history of the syndrome, implying a high risk of readmission and death.^4-6^ These data have been partially reproduced in studies in Brazil and are observed in the Brazilian public system statistics.^2,7^ The recent publication of the initial results of the BREATHE Registry, which included 52 centers in Brazil, clearly demonstrates the great impact of the syndrome, with in-hospital mortality of 12,6%.^8^

Despite the importance of the BREATHE Registry for Brazil, the majority of existing cohorts of acute decompensated HF were performed in the United States or Europe, including patients with a clinical, etiological, social and economic profile different from that of Brazilian patients.^9^ In addition, an aspect not explored in the scenario of hospitalized patients refers to the hospital and extra-hospital evolution of patients hospitalized for decompensated HF compared to the prognosis of patients hospitalized for other causes, but who present a previous diagnosis of HF. It is plausible to speculate that the presence of HF, even if this is not the primary cause of hospitalization, implies a reserved prognosis. In this context, it is still of great value to recognize prognostic predictors in order to identify patients requiring more intensive monitoring and treatment.^10^ The objective of the present study is to identify the predictors and causes of in-hospital mortality in patients hospitalized for acute decompensated HF compared to those who have HF and hospitalize for other conditions in a Brazilian public tertiary hospital.

## Methods

### Location, design and patients

This study was carried out in a public hospital of tertiary level in Porto Alegre, Rio Grande do Sul, Brazil, with approximately 850 beds. This is a prospective cohort study, in which adult patients (≥ 18 years) were admitted to any ward or intensive care unit (ICU) of this hospital and who were identified as having HF as indicated by the attending physician in the score of Charlson's comorbidity, or simply Charlson's index, via electronic medical records. Were excluded from the analisys pediatric patients (age <18 years), with permanence only in the emergency department (without admission to the infirmary or in the ICU), with hospital evasion or unavailable computerized discharge.

In this hospital, the Charlson index is filled out by the attending physician in the electronic medical record in a compulsory manner at the time of admission and at discharge. Failure to complete it prevents continuity of diagnostic and therapeutic procedures or hospital discharge. Although it was developed to predict risk in patients admitted to elective surgical procedures, the Charlson index has been described as an excellent tool for hospital use for clinical prediction of in-hospital mortality.^11^ It is a score composed of several comorbidities that is widely used to classify the severity of patients, and it is possible to compare the burden of diseases of patients from different medical and hospital services. The comorbidities that make up the Charlson index are acute myocardial infarction (AMI), congestive heart failure, peripheral and aortic vascular disease, cerebrovascular disease, dementia, chronic obstructive pulmonary disease (COPD), connective tissue disease, ulcer disease, moderate to severe kidney disease (creatinine > 3.0 mg/dL), hemiplegia, lymphoma/myeloma, leukemia/polycythemia vera, tumor, AIDS, and metastatic cancer.^12^

### Logistics and data collection

For purposes of analysis, patients who had multiple hospitalizations had considered only their last hospitalization, so that all in-hospital deaths of the sample were included, avoiding that more severe patients with multiple readmissions had their characteristics analyzed multiple times and seeking to preserve independence of the data. Data collection was performed by internal medicine residents previously trained through the standardized review of electronic medical records, and a computerized collection protocol was created and fully integrated into the electronic medical record of the hospital. 10% of the sample was verified by two other researchers of the study, preceptors of the Internal Medicine Service, to measure the reliability of the data collected. The patients were selected through a computerized system that allowed the automatic identification of all those who fulfilled the inclusion criteria. The causes of in-hospital death were stratified into cardiovascular death (HF, acute coronary syndrome, stroke or other cardiovascular deaths) and non-cardiovascular death (from infection, neoplasia, respiratory origin or other non-cardiovascular death). When the collectors could not identify the cause of mortality, the case was evaluated by two experienced researchers. If they could not identify the cause of death, it was defined as "death for an indefinite cause."

The following variables and instruments were included in data collection: age; sex; geographical origin; (Porto Alegre, Metropolitan Region of Porto Alegre and interior); team in which the patient was hospitalized (Cardiology, Internal Medicine and Others); length of hospital stay; cause of hospitalization; Charlson index; laboratory values (urea, sodium, creatinine and potassium) in the first 24 hours of hospitalization; echocardiographic data up to 1 year before admission: left ventricular ejection fraction (LVEF); left ventricular hypertrophy; presence of diffuse hypokinesia or segmental alterations of contractility and valvular lesions; prescription of cardiovascular drugs at hospital discharge; non-pharmacological guidelines at hospital discharge; outpatient referral; hospitalization in ICU, intra-hospital death; reason for in-hospital death; emergency visit and rehospitalization within 30 days after hospitalization.

The sample was separated into two groups: patients who had heart failure and hospitalized for a reason other than acute decompensated HF (NDHF) and patients who had acute decompensated acute HF (ADHF) as the reason for hospitalization. The latter group consisted of patients who presented as the main diagnosis, defined by the attending physician, one of the International Codes of Disease (ICD) presented in Appendix 1. According to Steinberg et al.,^13^ we stratified the patients into three subgroups of LVEF: preserved LEVF (> 50%), borderline LVEF (40-49%) or reduced LVEF (<40%).

### Data analysis

Continuous variables were expressed as mean and standard deviation or median and interquartile range (IIQ 25% -75%) according to normality of data analyzed using the Shapiro-Wilk test. Categorical variables were expressed as frequency and percentages. Univariate analyzes were performed by unpaired Student's t test, Mann-Whitney test, Poisson's test and chi-square test. For the multivariate analyzes, the Poisson regression with estimation of robust variances was performed by the stepwise methodology, calculating the incidence ratios and the 95% confidence intervals. From the data collected from the patients, univariate analyzes of continuous and categorical variables were performed within each of the two pre-defined groups (ADHF and NDHF). The variables that had a value of p < 0.20 in the univariate analysis were selected for the multivariate analysis in order to identify the predictors of in-hospital mortality. A p value of 5% was considered statistically significant. Due to the potential multicollinearity effect, two models of statistical analysis were used, one with a Charlson index and urea (model 1), but without the items that make up the Charlson index (comorbidities and age) and the other without the Charlson index and urea (model 2). The accuracy of both methods was similar. The data collected in the computerized system customized for the research were exported to a Microsoft Excel version 18 spreadsheet (*Microsoft Inc*., Redmond, USA) and the statistical analyzes were conducted by the Statistical Package for the Social Sciences (SPSS) Basic version 19.0 (SPPS Inc., Chicago, USA).

The research project was approved by the Research Ethics Committee of the institution of the corresponding author. There were no sources of study funding.

## Results

### Patients

All patients admitted to the hospital from January 1, 2009 to December 31, 2010, and had congestive heart failure as one of the diseases fulfilled in the Charlson index (compulsory filling in the patient's hospitalization), totalizing 2056 patients and 2666 hospitalizations were included. The characteristics of the patients in the sample are listed in table 1. In the population studied, the distribution between the sexes was homogeneous, the median age of the patients was 71 years, and the majority of the patients came from Greater Porto Alegre (59.3%) and was admitted under the care of the Cardiology (37.8%) and Internal Medicine (29%) teams. The median length of hospital stay was 15 days (IIQ 25-75%: 10-23) and the Charlson index was 5 (IIQ 25-75%: 4-7). Only 590 patients (28.7%) were hospitalized for acute decompensated HF. We observed throughout our sample that 43.1% of the patients had reduced LVEF, 18.9% had borderline LVEF and 38% had preserved LVEF. When we analyzed, however, the two subgroups of patients, we found that the patients who hospitalized for ADHF had a higher prevalence of reduced LVEF compared to patients hospitalized for other reasons (58% X 36.3% respectively), similar prevalences of LVEF (17.4% X 19.6% respectively) and lower percentage of preserved LVEF (24.6% X 44.1% respectively).

**Table 1:** Baseline characteristics of ADHF and NDHF patients

	All (n = 2056)	ADHF (n = 590)	NDHF (n = 1466)	p value
Age (years)	71 (61 - 79)	70 (60 - 79)	71 (61 - 80)	0,11
Male	1041 (51%)	301 (51%)	740 (50%)	0,81
White Race	1736 (84%)	490 (83%)	1246 (85%)	0,25
Length of stay (days)	15 (10 - 23)	13 (9 - 20)	16 (10 - 24)	< 0,001
VE ejection fraction (%)	44 (36 - 59)	38 (31 - 49)	47 (40 - 64)	< 0,001
Charlson index	5 (4 - 7)	5 (4 - 7)	6 (4 - 7)	< 0,001
ICU hospitalization	362 (18%)	88 (15%)	274 (19%)	0,041
Cerebrovascular disease	361 (18%)	65 (11%)	296 (20%)	< 0,001
Previous AMI	503 (24,5%)	114 (19%)	389 (26,5%)	0,001
Diabetes mellitus	646 (31%)	171 (29%)	475 (32%)	0,13
Kidney disease*	287 (14%)	81 (14%)	206 (14%)	0,83
Peripheral vascular disease	307 (15%)	60 (10%)	247 (17%)	< 0,001
Neoplasia	49 (2 %)	6 (1%)	43 (3 %)	< 0,01
COPD	472 (23%)	106 (18%)	366 (25%)	0,001
Dementia	174 (8,5%)	41 (7%)	133 (9%)	0,12
Liver disease	86 (4%)	29 (5%)	57 (4%)	0,33
Urea (mg / dL) †	56 (42 - 78)	55 (42 - 79)	56 (42 - 78)	0,41
Creatinine (mg/dL)†	1,21 (0,94 - 1,59)	1,20 (0,98 - 1,56)	1,21 (0,92 - 1,60)	0,74
Sodium (mg/dL)†	138 (136 - 140)	139 (136 - 141)	138 (136 - 140)	< 0,001
Potassium (mEq/L)†	4,4 (4,0 - 4,8)	4,3 (4,0 - 4,8)	4,4 (4,1 - 4,8)	0,02

Data expressed in absolute number and percentage, except if indicated. Continuous values expressed as median and interquartile range; Student's t test, Mann-Whitney test or chi-square test were used for statistical analysis as indicated. ADHF: acutely decompensated heart failure; NDHF: patients with non-decompensated heart failure admitted for non-HF conditions; LV: left ventricle; ICU: intensive care unit; AMI: acute myocardial infarction; COPD: chronic obstructive pulmonary disease;

* Defined by creatinine > 3.0 mg/dL;

† Laboratory values within the first 24 hours of admission.

### Causes of death

During admission, 361 (17.6%) patients died, with a 19% mortality rate in the ADHF group and 17% mortality in the NDHF group. Table 2 shows the causes of death stratified by the two groups of analysis. It was found that approximately 60% of the cases of mortality were attributed to non-cardiovascular causes in the studied population, being higher in the group of patients with NDHF (63.6% versus 47.4%, p = 0.004). Of non-cardiovascular deaths, the most common cause in both groups was infection related, accounting for one-third of total deaths in the ADHF group and approximately half of all deaths in the NDHF group. On the other hand, death due to cardiovascular causes was more prevalent in the ADHF group (42.1% versus 28.7%, p = 0.016). Interestingly, in both groups, deaths attributed to heart failure occurred in only 21.6% of deaths in the study population, being more frequent in those patients with ADHF.

**Table 2:** Causes of intra-hospital deaths in the groups of patients with ADHF and NDHF

	Todos (n = 361)	ADHF (n = 114)	NDHF (n = 247)	p Value
CV death	119 (33%)	48 (42,1%)	71 (28,7%)	0,016
Heart failure	78 (21,6%)	39 (34,2%)	39 (15,8%)	< 0,001
Acute coronary syndrome	17 (4,7%)	4 (3,5%)	13 (5,3%)	0,60
Stroke	9 (2,5%)	3 (2,6%)	6 (2,4%)	1,000
Other CV causes	15 (4,2%)	2 (1,8%)	13 (5,3%)	0,16
Non-cardiac death	211 (58,4%)	54 (47,4%)	157 (63,6%)	0,004
Infection	159 (44%)	38 (33,3%)	121 (49,0%)	0,006
Neoplasia	6 (1,7%)	1 (0,9%)	5 (2%)	0,67
Respiratory Cause	18 (5%)	7 (6,1%)	11 (4,5%)	0,60
Other non-CV causes	28 (7,8%)	8 (7,0%)	20 (8,1%)	0,83
Undefined or ill-defined cause of death	31 (8,6%)	12 (10,5%)	19 (7,7%)	0,42

The chi-square test was used for statistical analysis. ADHF: acutely decompensated heart failure; NDHF: patients with non-decompensated heart failure admitted for non-HF conditions.

### Univariate and multivariate analysis in the NDHF group

Table 3 describes clinical characteristics that were associated with in-hospital mortality in the group of patients with NDHF. On univariate analysis, the following variables were identified as predictors of risk: age, length of stay, Charlson index, serum potassium and urea levels, and presence of clinical comorbidities. Admission in a Cardiology team had a small protective effect. In the multivariate analysis, independent risk predictors were: age, length of stay, presence of renal disease and dementia (Table 4). When included in the analysis (model 1), the Charlson index was also an important predictor of risk of in-hospital mortality, with moderate-severe renal disease being the comorbidity with the greatest magnitude.

**Table 3:** Univariate analysis of mortality predictors in the NDHF group

Predictors	RR (IC 95%)	p value
Age	1,028 (1,019 - 1,038)	< 0,0001
Lenght of stay	1,007 (1,004 - 1,010)	< 0,0001
Charlson index	1,185 (1,147 - 1,223)	< 0,0001
Creatinine	1,006 (0,974 - 1,040)	0,709
Potassium	1,209 (1,049 - 1,393)	0,009
Urea	1,004 (1,002 - 1,005)	< 0,0001
VE ejection fraction	0,993 (0,985 - 1,003)	0,126
Cardiology team	0,909 (0,868 - 0,952)	< 0,001
Ejection fraction VE ≤ 40%	1,157 (0,904 - 1,480)	0,248
Solid neoplasia	1,835 (1,148 - 2,932)	0,011
Dementia	2,412 (1,860 - 3,128)	< 0,001
Cerebrovascular disease	1,820 (1,437 - 2,304)	< 0,001
Kidney disease	2,610 (2,076 - 3,282)	< 0,001
Peripheral and aortic vascular disease	1,218 (0,920 - 1,614)	0,169
Liver disease	1,482 (0,926 - 2,371)	0,101

Poisson test was used for statistical analysis. NDHF: patients with non-decompensated heart failure admitted for non-HF conditions; RR: relative risk; 95% CI: 95% confidence interval; LV: left ventricle.

**Table 4:** Multivariate analysis of mortality predictors in the NDHF group

Predictors	Model 1* (Including CCI)		Model 2† (Excluding CCI)	
	RR (IC 95%)	p value	RR (IC 95%)	p value
Age	NA	NA	1,003 (1,002 - 1,005)	< 0,001
Lenght of stay	1,002 (1,000 - 1,003)	0,036	1,002 (1,000 - 1,003)	0,018
Charlson index	1,030 (1,019 - 1,041)	< 0,0001	NA	NA
Cardiology team	0,994 (0,949 - 1,040)	0,780	0,999 (0,959 - 1,043)	0,988
Urea	1,000 (1,000 - 1,001)	0,113	NA	NA
Potassium	1,011 (0,980 - 1,043)	0,482	1,012 (0,981 - 1,045)	0,445
Neoplasia	NA	NA	1,111 (0,923 - 1,338)	0,267
Cerebrovascular disease	NA	NA	1,056 (0,995 - 1,121)	0,071
Peripheral and aortic vascular disease	NA	NA	1,060 (0,984 - 1,141)	0,126
Kidney disease	NA	NA	1,206 (1,115 - 1,304)	< 0,001
Dementia	NA	NA	1,176 (1,078 - 1,283)	< 0,001
Ejection Fraction VE ≤40%	1,028 (0,984 - 1,075)	0,219	1,032 (0,989 - 1,077)	0,151

CCI: Charlson's comorbidity index; RR: relative risk; 95% CI: 95% confidence interval; NA: not applicable; LV: left ventricle;

* Result after withdrawal of solid neoplasm, cerebrovascular disease, renal disease, peripheral and aortic vascular disease, dementia, liver disease, neo hematological disease, pulmonary disease, acute myocardial infarction and age by potential multicollinearity effect with CCI;

† Outcome after CCI withdrawal and urea due to potential multicollinearity effect with the above comorbidities.

### Univariate and multivariate analysis in the ADHF group

Table 5 describes clinical characteristics that were associated with in-hospital mortality in the group of ADHF patients. Mortality predictors were: age; length of stay; Charlson index; serum levels of sodium, potassium, urea and creatinine; And the presence of clinical comorbidities that make up the Charlson index. Admission in a cardiology team had a small protective effect. In the multivariate analysis, independent predictors of risk were: age, changes in urea and potassium levels, presence of renal disease, dementia, AMI and neoplasia (Table 6). When included in the analysis (model 1), the Charlson index was also a predictor of in-hospital mortality risk, with moderate-severe renal disease being the most relevant comorbidity, since the number of patients with neoplasia was very small (n = 6).

**Table 5:** Univariate analysis of the predictors of mortality in the ADHF group

Predictors	RR (CI 95%)	p value
Age	1,025 (1,01 - 1,04)	< 0,001
Lenght of stay	1,006 (1,001 - 1,011)	0,012
Charlson index	1,280 (1,215 - 1,348)	< 0,0001
Potassium	1,470 (1,235 - 1,751)	< 0,0001
Urea	1,010 (1,007 - 1,012)	< 0,0001
Creatinine	1,168 (1,047 - 1,302)	0,005
Sodium	0,955 (0,867 - 0,999)	0,048
Ejection fraction VE ≤ 40%	0,999 (0,712 - 1,402)	0,996
Cardiology team	0,931 (0,872-0,994)	0,033
Neoplasia	4,488 (3,021 - 6,668)	< 0,0001
Dementia	2,693 (1,847 - 3,925)	< 0,0001
Cerebrovascular disease	2,400 (1,685 - 3,418)	< 0,0001
Kidney disease	3,687 (2,732 - 4,976)	< 0,0001
Peripheral and aortic vascular disease	2,369 (1,648 - 3,404)	< 0,0001
Liver disease	1,667 (0,943 - 2.946)	0,079
AMI	1,786 (1,264 - 2,522)	0,001
COPD	1,550 (1,075 - 2,234)	0,019

Poisson test was used for statistical analysis. ADHF: acute compensated cardiac insufficiency; RR: relative risk; 95% CI: 95% confidence interval; LV: left ventricle; AMI: acute myocardial infarction; COPD: chronic obstructive pulmonary disease.

**Table 6:** Multivariate analysis of the predictors of mortality in the group of patients hospitalized for acute HF (ADHF)

Predictors	Model 1* (Including CCI)		Model 2† (Excluding CCI)	
	RR (CI 95%)	p value	RR (CI 95%)	p value
Age	NA	NA	1,002 (1,000 -1,004)	0,004
Lenght of stay	0,996 (0,99 - 1,001)	0,99	0,999 (0,99 - 1,003)	0,821
Charlson index	1,034 (1,02 - 1,05)	< 0,0001	NA	NA
Urea	1,001 (1,000 -1,002)	0,014	NA	NA
Sodium	0,998 (0,992 - 1,005)	0,595	0,996 (0,990 -1,002)	0,238
Potassium	1,042 (1,006 -1,079)	0,021	1,036 (1,003 - 1,070)	0,032
Cardiology team	0,966 (0,912 - 1,023)	0,243	0,969 (0,92 - 1,02)	0,266
Kidney disease	NA	NA	1,22 (1,12 - 1,33)	< 0,001
Dementia	NA	NA	1,152 (1,03 - 1,29)	0,014
Neoplasia	NA	NA	1,373 (1,01 - 1,87)	0,044
Cerebrovascular disease	NA	NA	1,051 (0,96 - 1,15)	0,291
Peripheral and aortic vascular disease	NA	NA	1,074 (0,97 - 1,18)	0,143
AMI	NA	NA	1,081 (1,01 - 1,16)	0,021
COPD	NA	NA	1,022 (0,95 - 1,10)	0,552

CCI: Charlson's comorbidity index; RR: relative risk; 95% CI: 95% confidence interval; NA: not applicable; AMI: acute myocardial infarction;

* Outcome after withdrawal of solid neoplasm, cerebrovascular disease, renal disease, peripheral and aortic vascular disease, dementia, liver disease, neo-hematological disease, lung disease, AMI, creatinine and age by potential effect Multicollinearity with CCI

† Outcome after withdrawal of CCI, urea and creatinine due to the potential effect of multicollinearity with the comorbidities and exams above.

## Discussion

Heart failure has been the subject of extensive research regarding the mortality and quality of in-hospital care. Most evidence evaluates patients with HF who hospitalizes for acute decompensation, identified by the primary diagnosis of discharge.^9^ However, the literature demonstrates that most patients with HF is admitted due to other causes.^14-18^ While quality measures of HF care are reported only in patients hospitalized for HF, some measures appear to be beneficial for all HF patients, regardless of the cause of hospitalization.^9,19,20^ In this study, we identified that in-hospital mortality was extremely high in both the groups; (1) age, (2) renal function and potassium levels (3) length of stay, and (4) burden of comorbidities were independent predictors of risk of death within the hospital.

When comparing NDHF patients with ADHF patients, we found that the firsts had more comorbidities, with higher Charlson index and LVEF values, similar to those found in the scientific literature.^14,15^ We found a prolonged lenght of stay when compared to hospitals in the USA^21^ and in Brazil itself,^5^ with the NDHF group having the highest median (16 days versus 13 days), which has already been described in other articles.^16,17,19^ We found an association between longer lenght of stay and greater mortality in the NDHF group, a result that has also been reproduced in other scenarios.^19,21,22^ One of the possible explanations for the above data is that the need for hospitalization due to causes not related to HF delimits patients with greater burden and severity of diseases, generating greater complexity of care. Another important issue is that exacerbation of comorbidities such as COPD and chronic renal failure may directly contribute to worsening severity of HF and compromising subsequent treatments and outcomes.^15^

Regarding mortality, the hospital death rate of the entire sample was 17.6%, considering the last hospitalization of the patients in the study period. This value is much higher than that found in other countries, and even in other Brazilian hospitals.^2,5-8,19^ Although there were 19% deaths in the ADHF group and 17% in the NDHF group, this difference was not statistically significant. Blecker et al.^18^ showed that there was a similar mortality rate at 1 year follow-up for ADHF and NDHF (25.6% versus 26.2%, respectively, p = 0.76). We believe that the differences found between our cohort and the international scenario may have been influenced by the organization of the Brazilian health system, by the patients' clinical characteristics and by cultural aspects related to end of life care. Thus, it would be hasty to attribute this result exclusively to the idiosyncrasies of the health system and variations in the management of the disease.

The analysis of the causes of death in the hospital environment showed that approximately 60% of the cases of mortality were attributed to non-cardiovascular causes, with a higher percentage in the group of patients with NDHF. Deaths attributed to HF occurred in only 21.6% of the sample, being more frequent in patients with ADHF. It is noteworthy that, even in the ADHF group, almost half of the patients died from non-cardiac causes - 33% from infectious causes - a similar number of HF-related death. Few studies have reported the causes of in-hospital deaths in patients with HF. In a study with 18 institutions in Thailand with ADHF patients (Thai ADHERE),^23^ there was 5.5% of in-hospital deaths (29% from infection, 27% from HF and 13% from acute coronary syndrome). Finally, a CHARM^15^ sub-study evaluated the mortality rate according to the primary diagnosis of hospitalization and found that stroke, HF and AMI were the most relevant causes of death in patients hospitalized for cardiovascular conditions. Among the group admitted for non-cardiovascular disease, the leading causes of death were cancer, lung disease and kidney disease. We did not find research that investigated the causes of hospital deaths in the NDHF group.

Regarding the predictors of in-hospital mortality, moderate-severe renal disease (creatinine> 3.0 mg/dL) was the main predictor of mortality in both groups and elevated serum urea in the first 24 hours of hospitalization only in the ADHF group, as demonstrated in other studies.^6,21,24,25^ In a North American study (ADHERE) with almost 120 thousand patients, there was a high prevalence of renal failure in patients with ADHF, with a great impact on hospital mortality.^25^ Elevated potassium at hospital admission was also an independent predictor in the ADHF group and was used in a composite score (APACHE-HF) that was able to adequately predict adverse events in ADHF patients.^26^ In a cohort of 122,630 patients from *Medicare*, the comorbidities most related to deaths in patients with HF were COPD, chronic renal failure, and acute renal failure.^27^ Dementia was also a relevant independent predictor of mortality in both groups of our sample, a fact also described in a cohort of 282 elderly.^15,28^ In our patients, age, as well as previous research,^6,10,29,30^ also proved to be a marker of risk. The actual magnitude of the presence of neoplasia in the prediction of risk of in-hospital death should be better studied in other cohorts, because their sample had low statistical power (n = 6 in the ADHF group).

Although not originally developed and tested to describe a *mix* of clinical patients, the Charlson index has been widely used to describe and adjust inpatient populations.^31,32^ In our study, with each increment of one point in the score, there was an increased risk of death in 3% in both groups. It should be noted that, although our patients are older than most of the individuals surveyed, this does not justify the higher burden of disease in our sample compared to the scientific literature. Similarly, in a cohort study in Canada of approximately 38,000 patients hospitalized for acute HF for the first time, the Charlson index was a good 30-day and 1-year mortality predictor, with values of 9.3% And 26% with a Charlson index of zero and 18.8% and 50.6% with a score ≥ 3.^33^ We also observed, as in this Canadian study,^33^ that previous MI was also a predictor of risk of death in patients with ADHF.

Although we have patients with characteristics different from those observed in the international literature, we found that most of the predictors of in-hospital mortality in our sample, which represents the Brazilian public real hospital world, are very similar to those previously published in other studies. In addition, although we assessed distinct populations, the predictors of in-hospital mortality found in both groups were very similar.

Hospitalization due to decompensated HF is an important variable related to mortality, although it accounts for less than a third of the total causes of hospitalization.^15,34^ The few studies comparing HF populations have shown that patients with NDHF do not receive the care that modify the prognosis of the disease.^14,16,17^ A study in which 4345 hospitalizations of patients with HF (39.6% ADHF) were evaluated, found that patients with NDHF had a 10% lower rate of prescription of angiotensin converting enzyme inhibitors and of angiotensin receptor blockers at hospital discharge in subjects with reduced LVEF and a 7% lower rate of LVEF assessment.^19^ In our sample, we identified that a substantial portion of hospital morbidity and mortality was related to patients hospitalized for secondary causes, presenting causes and predictors of death of relevance similar to those with ADHF. To date, in most hospital settings, there is a priority focus on HF management, which may divert attention to the treatment of other diseases that significantly affect subsequent outcomes.^35^ It is suggested that the evidenced based treatment for HF may improve the survival of patients with HF, regardless of the cause of hospitalization.^14,16,17,36^ In this context, inadvertently neglecting other comorbidities in HF patients may represent a loss of opportunity to reduce admissions, improve the care of HF and reduce overall costs with HF.^37^

The findings of this research should be evaluated through some limitations of our study design. First, we analyzed only the latest hospital admission of each patient, which is likely to have overestimated in-hospital mortality. However, since the main objective of the study was to identify causes and predictors of mortality, this methodology allowed to have all the deaths of the sample. Second, data from a Brazilian tertiary hospital are not representative of the entire country, and there may be a limitation in its generalization. Finally, it should be emphasized that, the analyses are based on registry data. Study results might be influenced by differences in disease assessment. On the other hand, all these data require compulsory electronic filling by the attending physician both at admission and at hospital discharge.

## Conclusion

Patients hospitalized with HF represent a high-risk group with high in-hospital mortality, regardless of the primary reason for hospitalization in a Brazilian tertiary hospital. Few deaths were attributed to HF and, in both groups, deaths from non-cardiovascular causes, mainly attributed to infections, prevailed. We identified that a substantial portion of hospital morbidity and mortality in HF patients was associated with hospitalizations due to secondary causes, and patients hospitalized for other reasons had similar predictors of death as those with ADHF. We observed that age, change in urea and potassium values, length of stay and comorbid burden were predictors of in-hospital mortality. These observations should call attention to opportunities to improve quality of care and reduce the costs associated with HF care, regardless of the cause of hospital admission, emphasizing the need for a more comprehensive management of both the HF and the associated comorbidities in patients with this pathology.

## Annex 1

**Annex 1:** International Codes of Disease (ICD) to identify cases of heart failure

Code	Description
I11.0	Hypertensive heart disease with congestive heart failure
I13.0	Hypertensive cardiac and renal disease with congestive heart failure
I13.2	Cardiac and renal hypertensive disease with congestive heart failure and renal insufficiency
I42.0	Dilated cardiomyopathy
I42.6	Alcoholic cardiomyopathy
I42.8	Other cardiomyopathies
I42.9	Cardiomyopathy, unspecified
I50.0	Congestive heart failure
I.50.9	Unspecified heart failure
J.81	Pulmonary edema, unspecified and other
